# Effects of neighbourhood and household sanitation conditions on diarrhea morbidity: Systematic review and meta-analysis

**DOI:** 10.1371/journal.pone.0173808

**Published:** 2017-03-15

**Authors:** Youngmee Tiffany Jung, Ryan James Hum, Wendy Lou, Yu-Ling Cheng

**Affiliations:** 1 Centre for Global Engineering and the Department of Chemical Engineering and Applied Chemistry, University of Toronto, Toronto, Ontario, Canada; 2 Dalla Lana School of Public Health, University of Toronto, Toronto, Ontario, Canada; George Washington University School of Medicine and Health Sciences, UNITED STATES

## Abstract

Sanitation in neighbourhood and household domains can provide primary protection against diarrhea morbidity, yet their distinct health benefits have not been succinctly distinguished and reviewed. We present here the first systematic review and meta-analysis of the distinct effect of neighbourhood and household sanitation conditions on diarrhea morbidity. We identified studies reporting the effect of neighbourhood-level exposure to wastewater or household sanitation facilities on diarrhea, by performing comprehensive search on five databases, namely the Cochrane library, PubMed, Embase, Scopus and Web of Science, from the earliest date available to February 2015. Twenty-one non-randomized studies and one randomized controlled trial met the pre-determined inclusion criteria, consisting of six datasets on neighbourhood sanitation conditions (total 8271 subjects) and 20 datasets on household sanitation (total 20021 subjects). We calculated the pooled effect estimates of neighbourhood and household sanitation conditions on diarrhea morbidity using the inverse variance random-effects model. The pooled effect estimates showed that both neighbourhood sanitation conditions (odds ratio = 0.56, 95%CI: 0.40–0.79) and household sanitation (odds ratio = 0.64, 95%CI: 0.55–0.75) are associated with reduced diarrheal illness, and that the magnitudes of the associations are comparable. Evidence of risk of bias and heterogeneity were found in the included studies. Our findings confirm that both neighbourhood sanitation conditions and household sanitation are associated with considerable reduction in diarrhea morbidity, in spite of a number of methodological shortcomings in the included studies. Furthermore, we find evidence that neighbourhood sanitation conditions is associated with similar magnitude of reduction in diarrhea morbidity as household sanitation. The findings suggest that, in addition to household sanitation provision, dual emphasis on neighbourhood sanitation through public sanitation infrastructure provision and community-wide sanitation adoption is advisable for effective reduction of diarrheal disease burden.

## Introduction

Sanitation is a primary barrier against fecal-oral transmission of pathogens that can cause diarrhea, which killed an estimated 0.5 million under-five children in 2015[1]. In addition to being ranked as the fourth leading cause of under-five child death[1], diarrhea morbidity in children has not decreased since the 1980s, persisting at 2 to 3 incidents per under-five child per year[2].

Diarrheal pathogens are transmitted within one’s domestic domain and the public domain, rendering household and public sanitary conditions crucial for diarrheal disease control[3]. Generally, household and neighbourhood sanitation conditions are improved through community-wide interventions for open defecation eradication and public fecal sludge and wastewater management infrastructure improvements. Recent community-led sanitation interventions, including the community-led total sanitation program[4] and India’s Total Sanitation Campaign [5], have placed emphasis on neighbourhood-wide uptake of household sanitation, for distinct protection against fecal pathogens in both household and neighbourhood domains [3,6]. However, the effectiveness of such community-wide interventions on preventing fecal pathogen transmission is unclear. UNICEF has reported that 70 to 80% of their previous community-led total sanitation attempts failed to achieve 100% sanitation coverage, in which case tangible reduction in neighbourhood-level fecal contamination and subsequent decline in diarrheal morbidity may not be attained[7–9]. Furthermore, a recent 12-city study by the Water and Sanitation Program[10] indicated that neighbourhood sanitation is compromised by poor fecal sludge and wastewater management infrastructure in the studied cities; in the study, the authors reported that, although 98% of urban households had access to household sanitation on average, 78% of the households discharged untreated fecal waste into the immediate environment without systematic management. Similar reports of indiscriminate wastewater discharge have been made in a number of Asian countries [11,12].

Understanding the distinct impact of neighbourhood and household sanitation conditions has significant implications for future sanitation intervention designs and related regulations[3]; yet, a systematic review of the relevant literature has not been conducted. Previous systematic reviews on the effect of sanitation on diarrhea morbidity have excluded much of the relevant literature due to inclusion restrictions on study design[13–18]. Most reviews exclusively included controlled trials[13,14,16,17] and observational studies with specific matching methods[15,18], of which only a limited number have assessed the effect of neighbourhood sanitation on diarrhea morbidity. A majority of the studies included in the previous reviews instead assessed the aggregated effects of neighbourhood sanitation, household sanitation, or other interventions (eg. water and hygiene). For instance, of the 13 studies reviewed by Clasen et al.[16], only two studies separately operationalized and analysed the effects of neighbourhood sanitation and household sanitation on diarrhea morbidity[19,20], while the rest of the studies reported aggregated effects. Studies that assessed the distinct effects of neighbourhood sanitation and household sanitation through other study designs have not been reviewed.

We present the first systematic review and meta-analysis of studies that distinguish and assess the distinct effects of neighbourhood sanitation and household sanitation conditions on diarrheal diseases. We define neighbourhood sanitation as the removal of exposed fecal matter or wastewater from the neighbourhood; we collectively review studies that make observation of exposed fecal matter or wastewater without distinguishing the underlying intervention, along with studies on specific interventions that ultimately remove exposed fecal matter or wastewater (eg. community-wide open defecation eradication and wastewater management infrastructure improvement). Household sanitation conditions is defined as the presence or use of household sanitation facility for child feces disposal.

The review is conducted adhering to the PRISMA (Preferred Reporting Items for Systematic Reviews and Meta-Analyses) statement[21] (S1 Table), and Meta-analysis Of Observational Studies in Epidemiology (MOOSE) guideline[22].

## Methods

### Search strategy and inclusion criteria

We searched the following databases from inception up to February 20 2015: the Cochrane library; PubMed; Embase; Scopus; and Web of Science. The search was performed using the following keywords: (sanitation OR toilet OR latrine OR excreta disposal OR sewer*) and (diarrhea* OR diarrhea OR diarrhoea* OR diarrhoea). Full search strategy is shown in S2 Table. Additionally, the references of the screened publications and review articles on the health effect of water, sanitation and hygiene were hand-searched for relevant articles. We did not apply any restriction on study design, study subject and location in our search. Abstracts, unpublished studies and non-English articles were excluded.

We reviewed eligible studies that analysed the correlation between diarrheal illness and neighbourhood and/or household sanitation conditions. We defined neighbourhood sanitation as the removal of exposed fecal matter or wastewater at the neighbourhood level. We included studies that measure neighbourhood sanitation by specific interventions such as improvement in sewerage and drainage or eradication of open defecation, and/or by observations such as absence of exposed wastewater, fecal matter, sewage spillage, or open drainage in the neighbourhood. Household sanitation conditions were defined as the presence of any type of household sanitation facility within the subject’s residence, or the disposal method of child feces. Studies that satisfied all of the following criteria were included exclusively: exposure factor is neighbourhood or household sanitation conditions, clearly distinguished from one another; outcome is individual’s incidence of diarrhea; effect size is reported in odds ratio (OR), relative risk (RR) or prevalence ratio (PR) with confidence intervals (CI); effect size is adjusted for confounding by potential confounders as per the judgment of the researchers. All study designs including observational studies were reviewed, to gain broad insight into the research question in shortage of evidence from neighbourhood sanitation intervention trials due to their inherent logistical complexity. We report the bias and differences in the included study designs following the Meta-analysis Of Observational Studies in Epidemiology (MOOSE) guideline [22]. To address heterogeneity across different study designs, we conducted subgroup analysis to differentiate the results between study designs where possible. We excluded neighbourhood sanitation studies that assessed community interventions of limited success (i.e., open defecation not eradicated), and household sanitation studies comparing the effect of different types of sanitation facilities (eg. pit latrine vs. flush toilet).

We exclusively included studies that quantified the distinct effect of neighbourhood or household sanitation through study design (eg. subject matching for household sanitation), or statistical adjustment (eg. multivariate regression separately parameterizing the effects of neighbourhood and household sanitation). Excluded studies were those that reported an aggregate measure of neighbourhood and household sanitation without controlling for sanitation status at the other level; for example, studies that assessed the effect of increased sewer connection without segregating the effect of improved household sanitation and neighbourhood conditions, were excluded.

If multiple publications were identified for the same clinical study, the most complete publication was used in our analyses. TJ performed initial screening of studies by titles and abstracts, and hand-searched the bibliographies of the screened studies to find additional studies. TJ and RH independently assessed the eligibility of the shortlisted studies based on the full texts. In case of disagreement, TJ and RH discussed the decisions until consensus was reached.

### Data extraction and risk of bias assessment

Relevant data were extracted and recorded by TJ and cross-checked by RH. The extracted data included author, year of publication, study design, population, location, period, description of study area, sample size, response rate, control group selection method, outcome measure, outcome measurement method, sanitation measure, definition of neighbourhood in case of neighbourhood sanitation measure, adjustment factors, adjusted and unadjusted effect size in OR, RR, or PR and their 95% CI.

TJ and RH independently assessed the risk of bias of the included studies, using a set of criteria suggested by Newcastle-Ottawa scale for observational studies [23] and the Cochrane Collaboration’s tool [24] for controlled trials, modified to reflect the design of the review and the nature of sanitation interventions as follows: (1) measured neighbourhood sanitation by a structured interview or field observation according to an objective definition of neighbourhood; determined presence and confirmed the use of household sanitation by a structured interview or field observation; (2) measured diarrhea outcome by clinical diagnosis, stool analysis, or a structured interview with diarrhea episode defined as more frequent passage of loose or liquid stools per day than normal, and a recall period of less than 2 weeks across all study subjects; (3) case-control studies selected controls from the same residential community as the case, are without recent history of diarrhea or similar diseases; cohort and non-randomized controlled studies selected comparison groups from the same subnational division for neighbourhood sanitation studies and from the same community for household sanitation studies; cross-sectional studies selected samples by random or multi-stage sampling, and randomized controlled trials selected intervention groups by sequence generation; (4) adjusted or matched for the key individual and household factors of child health[25], including age, socioeconomic status (e.g. parental education level, income), and one of either household water supply type or hygiene behavior; (5) case-control studies and cross-sectional studies have participation rate higher than 80%, and cohort, non-randomized controlled trials and randomized controlled trials have loss to follow-up less than 20%. The assessments were made descriptively without scoring, out of concern for the lack of validity and relevance in ad-hoc quality scales[22].

### Statistical analysis

The pooled effect estimate in OR was obtained using the inverse-variance method, the most commonly used pooling method which calculates a weighted average of the included effect sizes, each weighted by its variance[26]. In the case where multiple effect sizes were reported in a single study, the effect sizes were averaged before being pooled with other studies[27]. I^2^ was used to quantify the percentage of the effect size variability that is caused by statistical heterogeneity rather than by chance[28]. The inverse-variance random-effects method was used to account for the notable degree of heterogeneity in our included studies, under the assumption that the true underlying effect follows a normal distribution[29]. Subgroup analyses were performed to investigate the observed heterogeneity, based on the measure of sanitation and outcome, study location, type of study area, and adjustment for confounding by all of age, socioeconomic status, and one of water source or hygiene behavior variables; sensitivity analyses were also performed to test whether the meta-analysis results are influenced by the analytical method, namely the inverse-variance random-effects versus the inverse-variance fixed-effect methods, and adjustment for confounding[27]. Publication bias was assessed using a regression test proposed by Peters[30]. All analyses were performed using Review Manager 5.3[31] and R 3.1.2[32].

## Results

**Fig 1:**
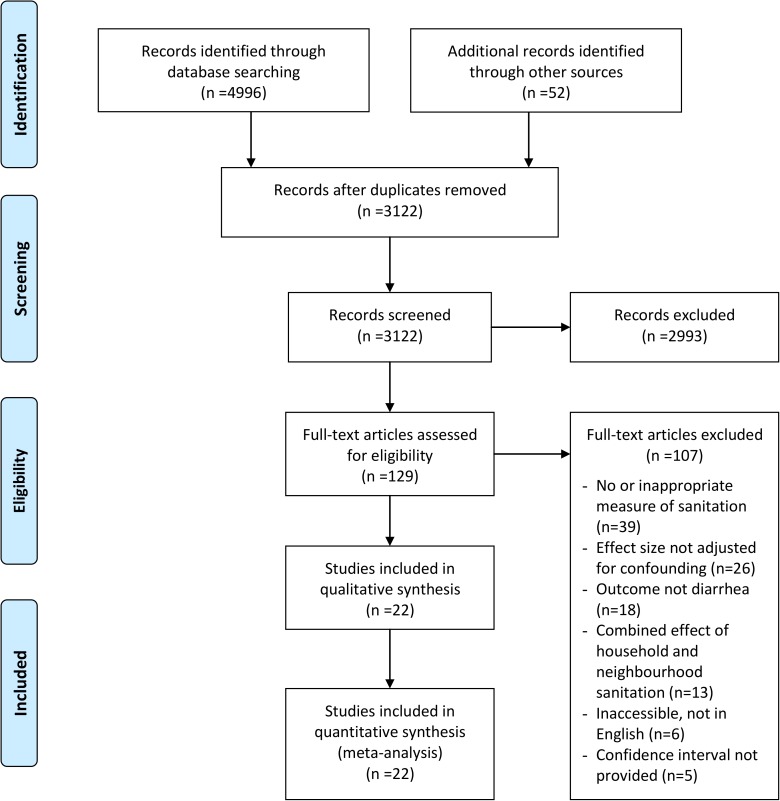
Flow diagram of study search and selection process.

Our search of databases and bibliographies of relevant literature yielded 5048 publications. Duplicates were removed, and the remaining publications were screened by the title and abstract, reducing the count to 129 (Fig 1). After a full-text review of the 129 publications, we obtained 22 eligible publications that met the inclusion criteria for our review. The studies that were included in the previous sanitation reviews[13–18,33], but aggregated the effects of household and neighbourhood sanitation, were excluded in this review.

We extracted three datasets from the publication by Anteneh et al.[34], (one dataset on neighbourhood sanitation and two on household sanitation), and two datasets from each of the publication by Agustina et al.[35] and Godana et al.[36]. We obtained one dataset from each of the other 19 publications. In total, 26 datasets from 22 publications were included in our meta-analyses, with six datasets from six publications on neighbourhood sanitation conditions (total 8271 subjects) and 20 datasets from 17 publications on household sanitation (total 20021 subjects). The two measures of household sanitation reported in the studies by Anteneh et al.[34], Agustina et al.[35] and Godana et al.[36] were averaged within each study prior to pooling, as suggested by Borenstein et al.[27]. The study designs of the included publications, as summarized in Table 1, were case-control (n = 9), cross-sectional (n = 8), cluster controlled trials (n = 3), or longitudinal (n = 2). The studies were conducted between 1984 to 2013 in 18 low-middle income countries in Africa, South America and Asia, in both rural (n = 10), urban (n = 9), or both rural and urban settings (n = 3). Primary study subjects were children under two, three, or five years of age (n = 20) except three publications that included older study subjects (7–12 years old[37]; up to 10 years old[38]; all age[39]). All included studies measured the incidence of diarrhea as the outcome. All authors reported the effect size in OR, except Heller et al.[40] and Clasen et al.[41], who reported the effect size in RR equivalent to OR and PR close to OR, respectively. Evidence for risk of bias was found in 17 of 22 included publications based on our assessment (Table 1) by a set of criteria described in the Methods section.

**Table 1:** Characteristics of studies included in the meta-analysis of the association of diarrhea morbidity with neighbourhood sanitation.

Reference	Study design	Study location, period	Subjects (#, age)	Diarrhea outcome	Exposure	Risk of bias assessment^a^^,^^b^				
						E	O	S	A	P
**Neighbourhood sanitation**										
Al-Ghamdi 2009[37]	Cross-sectional	Urban, Saudi Arabia, 2004–2005	1064 (7–12 yrs)	Incidence 1-month recall (yes/no)	No sewage spillage around house (yes/no*)	n	n	n	n	n
Anteneh & Kumie 2010[34]^c^	Cross-sectional	Rural, Ethiopia, 2006	447 (<5 yrs)	Incidence 2-week recall (yes/no)	No observable feces in the neighborhood yard (yes/no*)	n	y	y	n	-
Ferrer 2008[38]	Case-control	Urban, Brazil, 2002–2004	3364 (<10 yrs)	Clinic admission for diarrhea	No open sewage ditch nearby(yes/no*)	n	y	y	y	-
Graf 2008[42]	Cross-sectional	Urban, Nairobi, 2006	717 (<5 yrs)	Incidence 2-week recall (yes/no)	No rubbish and fecal material lying around, blocked open drains around home and nearby streets(1 scale increment/4 scales)	n	y	y	n	-
Heller 2003[40]	Case-cohort	Urban, Brazil, 1993–1994	1996 (<5 yrs)	Physician diagnosis of diarrhea	No wastewater in street (yes/no*)	n	y	n	y	n
Moraes 2003[43]	Cohort	Urban, Brazil, 1980–1990	683 (<5yrs)	Incidence 2-week recall, more than twice expected number of episodes	Communities with simplified sewerage and surface drainage vs. surface drainage only*	y	y	y	y	y
**Household sanitation**										
Agustina 2013[35]^c^	Cross-sectional	Urban, Indonesia, 2004–2005	274 (12-59mths)	Defecation description 1-week record, categorized as diarrhea by field worker	Child feces disposal in latrine (yes/no*),. Presence of household latrine (yes/no*)	y	y	n	n	-
Anteneh & Kumie 2010[34]^c^	Cross-sectional	Rural, Ethiopia, 2006	447 (<5 yrs)	Incidence 2-week recall (yes/no)	Child defecation in latrine (yes/no*), Presence of functional household latrine (with sub & superstructure, providing service) (yes/no*)	y	y	y	n	-
Aziz 1990[19]	NRCT^d^	Rural, Bangladesh, 1984–1987	1359 (<5yrs)	Incidence 1-week recall (yes/no)	Child defecation or feces disposal in latrine	y	y	y	y	-
Baltazar 1989[44]	Case-control	Urban, Philippines, 1985	665 (< 2yrs)	Clinic admission for diarrhea	Child defecation or feces disposal in latrine (yes/no*)	y	y	n	n	y
Clasen 2014[41]^c^	CRCT^d^	Rural, India, 2010–2013	3835 (<5 yrs)	Prevalence 1-week record	Presence of functional household latrine (with roof; pan not broken; no hindrance for usage) (yes/no*)	y	y	y	y	y
Daniels 1990[45]	Case-control	Rural, Lesotho, 1987–1988	1613 (<5 yrs)	Clinic admission for diarrhea	Presence of household latrine (yes/no*)	y	y	y	y	y
Dessalegn 2011[46]	Cross-sectional	Rural/urban, Ethiopia, 2009	768 (<5 yrs)	Incidence 2-week recall (yes/no)	Presence of household latrine (yes/no*)	n	y	y	y	y
Dikassa 1993[47]	Case-control	Urban, Zaire, 1988	214 (<3 yrs)	Clinic admission for diarrhea	Child feces disposal in latrine (yes/no*)	y	y	y	y	y
Garrett 2008[20]	NRCT^d^	Rural, Kenya, 2001	960 (<5 yrs)	Incidence 1-week recall (yes/no)	Presence of household toilet (yes/no*)	y	y	y	y	n
Godana 2013[36]^b^	Case-control	Rural, Ethiopia, 2013	593 (<5 yrs)	Diarrhea incidence 2-week recall (yes/no)	Disposal of infant feces in latrine (yes/no*), Presence of household latrine (yes/no*)	y	y	y	n	y
Knight 1992[48]	Case-control	Rural, Malaysia, 1989	196 (4–59 months)	Clinic admission for diarrhea	Presence of household toilet (yes/no*)	y	y	n	y	y
Mbonye 2004[49]	Cross-sectional	Rural, Uganda, 2001	323 (<2 yrs)	Incidence 2-week recall (yes/no)	Presence of household pit latrine (yes/no*)	n	y	y	y	-
Mertens 1992[50]	Case-control	Rural, Sri Lanka, 1987–1988	3694 (<5 yrs)	Clinic admission for diarrhea	Child defecation in latrine or covered pit vs. open defecation*	y	y	n	y	n
Mihrete 2014[51]	Cross-sectional	Rural/urban, Ethiopia, 2012	925 (<5 yrs)	Diarrhea incidence 2-week recall (yes/ no)	Presence of household toilet (yes/no*)	y	y	y	y	-
Oketcho 2012[52]	Case-control	Rural, Tanzania, 2011	303 (<5 yrs)	Clinic admission for diarrhea	Child defecation in toilet or latrine (yes/no*)	y	y	n	y	-
Traore 1994[53]	Case-control	Urban, Burkina Faso, 1991	2793 (<3 yrs)	Clinic admission for diarrhea	Child feces disposal in latrine vs. elsewhere (yard, etc.)*	y	y	y	y	n
Tumwine 2001[39]	Cross-sectional	Rural/urban, East Africa, 1997	1015 (All age)	Incidence 1-week recall (yes/no)	Presence of household latrine (yes/no*)	y	y	y	n	-

a. E: Adequate measure of exposure O: Adequate measure of outcome; S: Appropriate sample and/or study group selection; A: Adjustment for child age, household socioeconomic status, and water or hygiene; P: Adequate participation or follow-up rate

b. y:yes; n:no;-:uncertain

c. Multiple datasets are extracted

d. NRCT: Cluster non-randomized controlled trials, CRCT: Cluster randomized controlled trials.

*Reference state.

**Fig 2:**
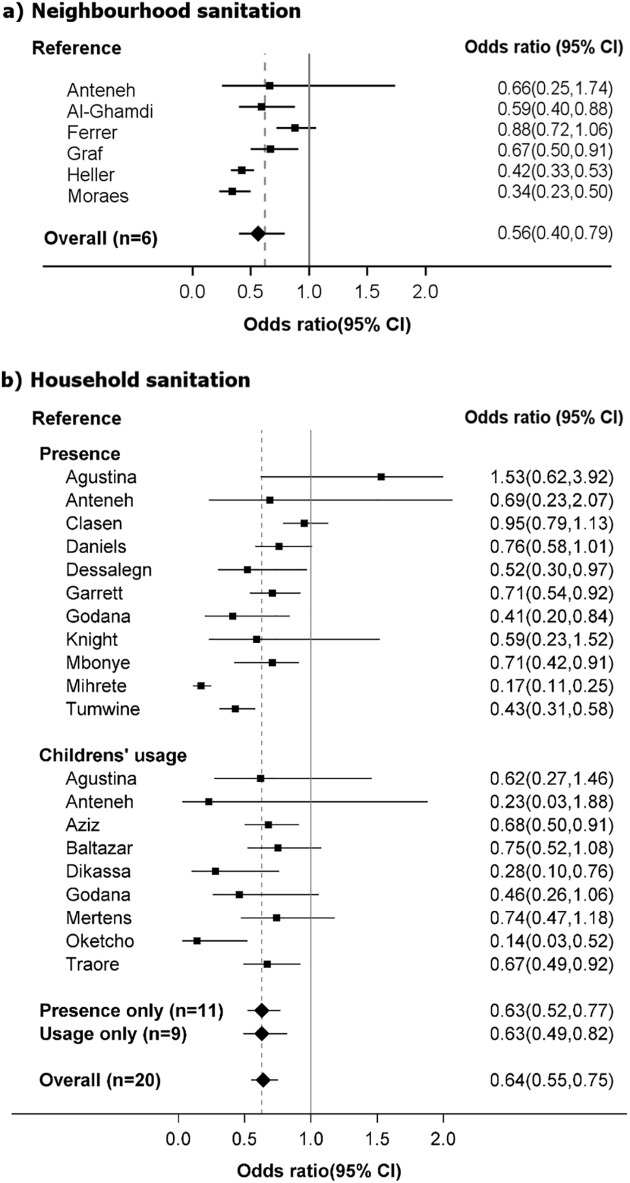
Meta-analysis of the association of diarrhea morbidity with a) neighbourhood sanitation (6 datasets) and b) household sanitation (20 datasets).

The pooled OR of neighbourhood and household sanitation on diarrhea morbidity were 0.56(95%CI = 0.40–0.79) and 0.64(95%CI = 0.55–0.75) (Fig 2), respectively.

Five of the six included neighbourhood sanitation studies measured the absence of visible wastewater or open drainage in the neighbourhood based on self-reports [34,37,38,40], by a 4-scale score based on assessment by the interviewer [42], without distinguishing the infrastructural sanitation intervention in place (e.g. fraction of households with improved sanitation, type of drainage system). Moraes et al., on the other hand, reported reduction in visible sewage in the neighbourhood that resulted from neighbourhood-wide reinforcement of public surface drainage with simplified sewerage system sanitation [43]. We did not identify any eligible studies on community-level sanitation coverage that met our inclusion criteria. All included studies controlled for potential confounding by household sanitation, through including a measure of household sanitation as a parameter in multivariate regression [34,38,40], or matching for household sanitation between the comparison groups[42,43,54], or finding absence of statistically significant correlation between household sanitation and diarrhea [37,41]. For instance, Moraes et al.[43] compared communities with similar distribution of household toilet types, but with different exposure to open sewage, as a result of community-level sewage collection systems. The geographic scale of neighbourhood was defined as politico-administrative neighbourhoods or villages[41,43,54] or more loosely as nearby or around household[37,38,42], neighbourhood yards[34] or as street without further elaboration[40]. Considerable between-study heterogeneity (I^2^ = 84%) was found in the reported effect sizes, but the direction of the effects were consistently protective without evidence of publication bias based on Peters regression test (p = 0.20)[30]. Due to limited number of studies, we performed subgroup analyses based only on outcome measure and adjustment for confounding[26].

Household sanitation was measured by the presence of a household sanitation facility (n = 11) and children’s use of a household sanitation facility (n = 9; direct defecation or disposal of child’s feces in a toilet) with three studies reporting both measures. Reports of household sanitation were based on either the interviewer’s observations (n = 4)[39,41,48,55], or the caretaker’s self-reports (n = 13)[19,34–36,44–47,49–53]. Anteneh et al.[34], Aziz et al.[19], Clasen et al.[41] and Garrett et al.[20] separately assessed the correlation between neighbourhood sanitation and diarrhea morbidity in multivariate analyses, while other studies adjusted for the subjects’ residential neighbourhood, by including community of residence as a parameter in a regression(n = 2), or by recruiting samples from the same community (n = 6), district or region (n = 5). The household sanitation coverage rate reported in 13 studies ranged from 10% to 90%. The between-study heterogeneity was moderate(I^2^ = 54%)[26], but the evidence of publication bias and small study effects was found in Peters regression test (p = 0.03)[30]. Cross-sectional studies and controlled trials reported significantly different association between household sanitation and diarrhea morbidity (p<0.05), but subgroup analyses based on the study location (Africa, Asia), type of study area (rural vs. urban), sanitation measure (presence or children’s use of household toilet), outcome measure (self-reported or clinic visit), and adjustment for confounding (full or partial), did not show significant difference in the association between household sanitation and diarrhea morbidity (p>0.05), as presented in Table 2.

**Table 2:** Summary of sensitivity and subgroup analyses.

	n^a^	Neighbourhood sanitation OR (95%CI)	n^a^^,^^b^	Household sanitation OR (95% CI)
**Overall**	6	0.56(0.40–0.79)	20	0.64 (0.55–0.75)
**Sensitivity analysis**				
Unadjusted effect sizes^c^	4	0.47(0.32–0.70)	12	0.52(0.42–0.65)
Fixed-effect method	2	0.61 (0.54–0.69)	20	0.69(0.63–0.76)
**Subgroup analysis**				
*Study design*				
Cross-sectional	-	-	8	0.51 (0.42–0.63)
Case control Cross-sectional	-	-	9	0.64 (0.53–0.77)
Controlled trials	-	-	3	0.81 (0.64–1.02)
*Study location*				
Africa	-	-	13	0.57(0.47–0.68)
Asia	-	-	7	0.86(0.75–0.99)
*Type of study ared*^*d*^				
Urban	-	-	5	0.72 (0.61–0.68)
Rural	-	-	12	0.69(0.51–0.92)
*Sanitation measure*				
Presence of household sanitation	-	-	11	0.63(0.52–0.77)
Children’s use of household sanitation	-	-	9	0.63(0.49–0.82)
*Outcome measure*				
Self-reported diarrhea	4	0.71 (0.61, 0.82)	13	0.63(0.50–0.80)
Clinic admission/ diagnosis of diarrhea	2	0.46 (0.38, 0.57)	7	0.68(0.55–0.83)
*Adjustment for confounding*^*e*^				
Fully adjusted	4	0.59 (0.37, 0.92)	12	0.67(0.57–0.80)
Partially adjusted	2	0.60 (0.51, 0.69)	8	0.57(0.40–0.81)

a.Number of datasets

b. Two datasets extracted from Agustina, Anteneh and Godana

c. Unadjusted effect sizes not reported in Graf, Kolahi, Garett, Knight, Mbonye, Aziz, Oketcho

d. Three studies on both rural and urban excluded

e. Adjustment forchild age, socioceonomic and water/hygiene.

## Discussion

Our meta-analyses showed that sanitary neighbourhood environment and household sanitation are independently associated with roughly 40% reduction of the odds of diarrhea morbidity, and that the magnitudes of the associations are comparable (neighbourhood sanitation OR(95%CI) = 0.56(0.40–0.79); household sanitation OR(95%CI) = 0.64(0.55–0.75)).

The observed protective direction of the association between diarrhea morbidity and neighbourhood and household sanitation are consistent with the previous reviews on the effect of sanitation on diarrhea morbidity, including the reviews by Esrey et al. (22% reduction)[13], Fewtrell et al. (RR(95%CI) = 0.68(0.53–0.87))[14], Clasen et al. (studies not pooled quantitatively, but generally protective)[16], Waddington et al. (RR(95%CI) = 0.63(0.43–0.93))[15] and Wolf et al. (RR(95%CI) = 0.72(0.59–0.88))[18]. Our findings are also in alignment with the reported association of sewer connection with diarrhea and enteric infection by Norman et al. (RR(95%CI) = 0·70(0·61−0·79))[56]. Though direct, quantitative comparison of the obtained effect sizes from this review and the previous reviews would be of interest, such comparison is not made here as the previous reviews included studies that do not differentiate the distinct effects of neighbourhood and household sanitation.

Despite the fact that most of the studies included in our review are observational in their design, the considerable magnitude of association between diarrhea and neighbourhood sanitation conditions, exclusive of household sanitation, is still compelling. Five [34,37,38,40,42] of the six included neighbourhood sanitation studies reported the protective correlation between absence of open wastewater and diarrhea, independent of household sanitation, which has been consistently reported in previous studies on diarrhea that met all our inclusion criteria except the effect estimate measure used (i.e., not OR, RR or PR), namely Genser et al.[57] and VanDerslice et al.[58]. Study by Moraes et al. [43] was the only study that assessed and reported the protective association of a distinguished intervention (i.e., improvement of sewerage) with diarrheal morbidity. Relevantly, Barreto et al. [59], excluded from this review based on effect measure used, also found correlation between improved sewerage connection and diarrheal morbidity. In addition, reduction in parasite infection was correlated with improved sewerage in Moraes et al.(2004) [60], under the same study as Moraes et al. (2003) [43], and Barreto et al. (2010)[61], under the same study as Barreto et al.(2007) [59]. It is worth noting that the study by Clasen et al.[41], the only randomized controlled trial included in this review on household sanitation, did not find significant impact of household sanitation on diarrhea morbidity. Not dismissing the protective effect of household sanitation, the authors attributed the null findings to persistent environmental fecal pathogens in the neighbourhood from insufficient increase in community-level fraction of households with access to functional household latrines (38% coverage at the end of the intervention), which may explain the null findings reported by other randomized controlled trials that also achieved marginal increase in sanitation coverage [9,62,63]. Indeed, a number of cross-sectional studies, mostly published as World Bank working paper, have found a significant correlation between increase in community level sanitation coverage and improved child health in terms of diarrheal illness[6,64], growth [65–67], and mortality [68]. The studies by Andres et al. [6] and Hunter and Prüss-Ütsün [64] were excluded from the review as the former study was published as grey literature, and the latter was published past the search period of our review. We found considerable similarity between the magnitudes of diarrhea morbidity reduction associated with neighbourhood sanitation and household sanitation, regardless of the analysis method (random effect or fixed effect) or the degree of adjustment for confounding.

### Heterogeneity

The between-study heterogeneity of the included studies was quantified using I^2^ statistic[26], which indicated that 84% and 54% of the variability in neighbourhood and household sanitation effect sizes, respectively, were caused by heterogeneity in the actual effect sizes rather than by chance. Subgroup analyses were performed to further investigate the source of the variability. The variability in the effect sizes of neighbourhood sanitation conditions was systematically associated with the outcome measure, but not with the degree of adjustment for confounders; subgroup analysis based on other factors, such as study design and location, was not conducted for neighbourhood sanitation studies due to the limited number of studies in each group. We found that the variability in household sanitation effect sizes is partially caused by varying study designs (cross-sectional vs. controlled trial), but not by study location, study area type, measure of sanitation or outcome, or the degree of adjustment for potential confounders. Variations in climate, social behavior, and adjustments of other potential confounders may have contributed to the heterogeneity. Based on the degree of heterogeneity observed, we primarily report on the pooled effects estimated by the random-effects model; similar pooled effect sizes were obtained by the fixed-effect model (Table 2).

### Limitations

The studies included in the review have several shortcomings. For instance, none of the included neighbourhood sanitation studies is a randomized controlled trial, due to the associated logistical complexity and high cost. In addition, a non-random pattern of sanitation self-selection may have been present in the included non-randomized studies; for example, participants with higher hygienic awareness may be more likely to adopt sanitation, in which case hygienic behavior can confound the association between sanitation and diarrhea morbidity. We attempted to control for such risk of non-random selection bias and confounding by exclusively pooling effect sizes adjusted for potential confounders in our meta-analyses and excluding unadjusted effect sizes that may exaggerate the effect estimate[69]; indeed, the pooled unadjusted effect sizes were marginally greater than pooled adjusted effect sizes in our sensitivity analysis (Table 2). Nonetheless, residual confounding may have still remained even after the adjustments, introducing bias to the calculated effect sizes and CIs. The direction and magnitude of such bias, however, cannot be estimated. Of relevance is the lack of direct adjustment for neighbourhood sanitation in 13 household sanitation studies, but the included studies controlled for the residing community (n = 8) or district and region (n = 5), thereby partially controlling for the potential confounding by neighbourhood sanitation.

Use of conventional blinding was not reported in any of the included studies, as it is not feasible in studies assessing the effect of sanitation interventions[16,33]. In addition, of the 22 included publications, 13 publications used self-reported diarrhea incidents, which may be biased with self-exaggeration or recall error[70]; however, the pooled effect of studies that used self-reported diarrhea incidents did not differ from those that used clinic admission due to diarrhea (Table 2). Recall bias is not likely to have been a significant issue, under the plausible assumption that the direction and magnitude of recall error are similar in intervention and control groups being compared.

Our search was limited to published studies on the basis that grey literature with unverified methodological features may introduce additional bias in our analyses, although published studies may selectively report the positive effect of neighbourhood and household sanitation over negative or null findings[26]. Among the excluded grey literature were World Bank policy working papers, of which a study by Andres et al. [6] met all other inclusion criteria for the review. The study confirmed the protective correlation between neighbourhood sanitation and child health. We estimated the likelihood of publication bias using a regression method developed by Peters[30], and found significant presence of small-study effects in household sanitation studies, but not in neighbourhood sanitation studies. The validity of Peters regression results, however, is unclear as it was performed on studies with considerable degree of heterogeneity[71], particularly in the case of neighbourhood sanitation studies.

We found considerable lack of details in reporting the degree of participation or follow-up and therefore could not gauge the magnitude of the risk of differential attrition with specific underlying causes. Of the 22 studies, only 13 studies reported the participation rate, of which five studies reported participation or follow-up below 80%. All controlled trials included [19,20,41] reportedly confirmed participant compliance during the follow-up phase by verifying the functionality and use of household latrines, but only Clasen et al. [41] reported the rate of compliance (36% of households with sign of present use).

Aside from the risk of bias associated with the included studies, a number of limitations were identified. The generalizability of our findings is limited by the lack of rigorous evidence across various contexts. We did not obtain sufficient information to explore neither the spillover benefit of household sanitation conferred on neighbours[6], nor the potential interaction between the role of household and neighbourhood sanitation, i.e., the degree to which the benefits of household and neighbourhood sanitation conditions are additional, or substitutional. The geographic scale of neighbourhood that is relevant to the risk of diarrhea morbidity remains unknown; neighbourhood scales were not clearly specified in some of the included studies, described as ‘around’ or ‘nearby’ households, or simply as ‘streets’.

## Conclusions

We present the first systematic review that investigates the distinct effects of neighbourhood sanitation conditions and household sanitation on diarrhea morbidity. The pooled effect estimates from our meta-analyses suggest that sanitary neighbourhood conditions and household conditions are both associated with reduced diarrheal burden, and that the magnitudes of the reduction are generally comparable. The finding is particularly relevant to the current lag in urban sanitation provision in developing countries, where a serious shortage of systemic fecal waste management has been reported at a neighbourhood level. Our findings imply that sanitation interventions should place dual emphasis on neighbourhood sanitation (e.g. improved public sewerage infrastructures) and household sanitation (e.g. private household toilets) to effectively reduce the risk of diarrhea. Further research evaluating the effect of neighbourhood and household sanitation utilizing rigorous measure of sanitation status and study design is desired to better inform sanitation intervention strategies and regulatory decisions.

## Supporting information

S1 Table PRISMA (Preferred Reporting Items for Systematic Reviews and Meta-Analyses) Checklist. (DOC)

S2 Table Full electronic search strategy. (DOCX)
