# Supplementary material for: Effects of neighbourhood and household sanitation conditions on diarrhea morbidity: Systematic review and meta-analysis
Source: PLoS One. 2017 Mar 15;12(3):e0173808. doi: 10.1371/journal.pone.0173808 (PMC5351971; doi:10.1371/journal.pone.0173808)
Supplement: S2 Table — (DOCX) [file pone.0173808.s002.docx]

# S2. Full electronic search strategy

| Database, Platform | PubMed, US National Library of Medicine |
| --- | --- |
| Search period | Date of inception to 2015/02/20 |
| Search strategy | Searched using following query for all fields, including MeSH terms for “sanitation”, “toilet facilities”, and “diarrhea”:  (("sanitation"[MeSH Terms] OR "sanitation"[All Fields]) OR toilet[All Fields] OR ("toilet facilities"[MeSH Terms] OR ("toilet"[All Fields] AND "facilities"[All Fields]) OR "toilet facilities"[All Fields] OR "latrine"[All Fields]) OR (excreta[All Fields] AND disposal[All Fields]) OR (sewer[All Fields] OR sewer's[All Fields] OR sewerage[All Fields] OR sewerage'[All Fields] OR sewerages[All Fields] OR sewerby[All Fields] OR sewered[All Fields]))  AND  (("diarrhoea"[All Fields] OR "diarrhea"[MeSH Terms] OR "diarrhea"[All Fields]) OR (diarrhea[All Fields] OR diarrhea'[All Fields] OR diarrhea''[All Fields] OR diarrhea's[All Fields] OR diarrhea,[All Fields] OR diarrheaal[All Fields] OR diarrheae[All Fields] OR diarrheal[All Fields] OR diarrheals[All Fields] OR diarrheas[All Fields]) OR (diarrhoea[All Fields] OR diarrhoea'[All Fields] OR diarrhoea's[All Fields] OR diarrhoea,[All Fields] OR diarrhoeae[All Fields] OR diarrhoeah[All Fields] OR diarrhoeal[All Fields] OR diarrhoeals[All Fields] OR diarrhoeas[All Fields] OR diarrhoeas'[All Fields])) |

| Database, Platform | Web of Science, Thomson Reuters |
| --- | --- |
| Search period | 1990 to 2015/02/20 |
| Search strategy | Searched using following query for Topic(TS) OR Title (TI):  TS=((sanitation OR toilet OR latrine OR excreta disposal OR sewer*)  AND (diarrhea* OR diarrhea OR diarrhoea* OR diarrhoea))  OR  TI=((sanitation OR toilet OR latrine OR excreta disposal OR sewer*)  AND (diarrhea* OR diarrhea OR diarrhoea* OR diarrhoea)) |

| Database, Platform | Scopus, Elsevier SciVerse  Cochrane Library, Wiley Online Library  Embase, Ovid |
| --- | --- |
| Search period | Date of inception to 2015/02/20 |
| Search strategy | Searched using following query for all fields:  (sanitation OR toilet OR latrine OR excreta disposal OR sewer*)  AND  (diarrhea* OR diarrhea OR diarrhoea* OR diarrhoea) |
